# Chicago Medical Society

**Published:** 1866-06

**Authors:** 


### CHICAGO MEDICAL SOCIETY.

Drs. R. M. Lackey, F. O. Earle and J. S. Sherman were duly elected members of the Society.

CEREBRO-SPINAL MENINGITIS.

Dr. Paoli read the history of an interesting case of this disease, successfully treated with bromide of potassium. The paper is given in full on another page of the Journal.

Drs. Bluthardt, Fitch, Clarke and Holmes, reported verbally cases in which the peculiar pain in the back of the head and neck, with more or less tonic contractions of the posterior cervical muscles were constant symptoms. In all these cases there were at some stage of the disease regular and marked exacerbations of the symptoms as if influenced by malaria. Quinine seemed to afford but temporary benefit.

### FATTY DEGENERATION OF KIDNEYS, ETC.

Dr. Bevan exhibited portions of the lungs, liver and kidneys of a patient who, after an illness of six weeks, had been brought to the Cook County Hospital three days before death. No satisfactory history of the case could be obtained. On entering the hospital, there were general anasarca, delirium and aphonia. The urine was found to contain large quantities of albumen. There was great dullness over the left lung.

The kidneys presented the peculiar mottled appearance, observed in several other specimens previously exhibited to the society, indicative of fatty degeneration.

The liver was atrophied and presented a marked hob-nail appearance.

The whole of the left lung was hepatized.

### URINARY CALCULUS.

Dr. Bogue exhibited a urinary calculus which he had removed from a child, three years of age, at the Cook County Hospital. Symptoms of stone had existed about a year. The calculus was of a very peculiar shape, long and pointed at each end, resembling almost perfectly a small almond. Its weight was 31 grains. A section through its short diameter showed marked concentric layers. It was principally composed of uric acid.

It was removed through the urethra, which had been previously dilated by means of dried sponge tent, and still further enlarged by small lateral incisions through the mucous membrane.

### OSTEO-MYELITIS.

Dr. Bogue also exhibited specimens of osteo-myelitis of the tibia and fibula following amputation, which had been performed in a case of frost bite. The patient, at the time of the exposure to cold, was in poor health in consequence of a severe gun-shot wound of the same limb. He did well for a few days, when he was seized with severe chills and fever. Symptoms of pyæmia soon appeared, followed by death in twelve days. An examination showed a large abscess in the left lung, which was extensively hepatized. The kidneys presented evident traces of fatty degeneration.

### ANEURISM OF THE AORTA.

Dr. Heydock exhibited a specimen of aneurism of the aorta, with the following most interesting history: Mr. B., captain of a lake vessel, æt. 35, had suffered for two years from aphonia and cough, with a degree of dyspnœa, which was partially relieved by bending forward. He had been examined by several careful and skillful practitioners. Patient came under the care of Dr. H. about four months ago. Pulse 74. There was constant pain in left arm and shoulder. The lungs were apparently healthy, except at the summit of the right side of the chest, where there were slight indications of tubercles.

No abnormal sounds of the heart could be detected by several experienced auscultators. One of the most marked and painful symptoms was extreme vigilance, which was scarcely affected by enormous doses of morphine.

At the autopsy which was recently made, the right lung was found perfectly healthy except at its summit, where there were small deposits of tuberculous matter.

The left lung was carnified and adherent in places to walls of the chest. Spleen healthy. There were calcareous deposits in the trachea. A large aneurism was found at the upper portion of the descending aorta, implicating the par vagum nerve. The recurrent laryngeal nerve seemed to have been destroyed by pressure from the aneurism.

### HARD CATARACT.

Dr. Holmes exhibited three specimens of hard cataract which he had recently extracted, and which were specially interesting as showing the capsule intact. In each case the extraction was preceded several weeks by a removal of a portion of the iris downwards. In extracting the two small specimens, after the section of the cornea had been completed, the opaque lens, covered with its capsule, at once escaped before the instrument for incising the capsule could be introduced. The pupil was thus left perfectly clear and free from fragments of the capsule and cortical substance. The recovery in these two cases was complete and most fortunate. By holding the small bottles to the light, the great delicacy and transparency of the capsule could be seen surrounding the opaque substance. In the case of the patient from whom was taken the specimen which seems much shriveled, the diagnosis could be readily made before the operation of a hard, yellowish, central cataract, floating in a cortical substance reduced to a milk-like fluid, since motions of the head caused the central hard nucleus to sink from one side to the other. The alcohol in which the specimen was preserved caused the fluid to pass through the capsule, leaving the latter in transparent folds.

In reference to the third specimen, Dr. Holmes stated that the case afforded a remarkable example of the manner in which an eye may suffer violence and yet recover sight. The patient was a lady, 66 years of age, and a cripple. The extraction was delayed six weeks after the iridectomy, in consequence of repeated attacks of diarrhœa and intermittent fever. After the recovery from the iridectomy, it was evident, the cataract was unusually large. Dr. H. stated, however, that he believed there was sufficient soft cortical substance to enable the cataract to pass through an opening of ordinary size in the cornea.

Although a large quantity of chloroform was administered, and the patient was so deeply under its influence as not to show signs of consciousness when the conjunctiva was seized with the forceps, and when the knife entered the cornea, yet, before the section was completed, the patient suddenly moved her head. This necessitated a second seizure of the conjunctiva with the forceps. With the most valuable aid rendered by his friend, Dr. Powell, during the operation, the section of the cornea, however, was completed without accident. After the rupture of the capsule with the usual instrument, it was found that the diagnosis had been faulty in regard to the condition of the cortical substance, the whole of which was very hard and quite firmly attached to the capsule. As the cataract had already entered the opening in the cornea, it was thought best to try gentle pressure on the globe rather than enlarge the incision in the cornea. After considerable (yet not undue) pressure, the whole lens escaped, the nucleus and hard cortical substance protruding from the capsule, like the pulp of a grape partially escaped from its skin. Not a trace of vitreous humor was lost.

A soft compress, soaked in a solution of atropine, was placed over the eye and confined by a bandage passed quite firmly around the head. At a visit late in the evening after the operation, the patient was very comfortable. Quite early in the night, however, she was attacked with severe diarrhœa. In spite of the attendants, she insisted on constantly getting out of bed.

Notwithstanding these unfortunate accidents, the cornea healed rapidly. Vision was excellent.

A comparison of the different specimens will show the unusual size of the cataract.

Dr. H. stated that, what with him occurred as an accident in removing the lens and capsule together, has been recommended by a recent writer as a measure advisable wherever it can be accomplished.

### REGULAR DISCUSSION OF THE EVENING.

### LOCOMOTOR ATAXY.

Dr. Heydock, in opening the discussion, stated that he would confine his remarks principally to the history of a single case now under his observation.

A gentleman, aged 61 years, having enjoyed good health, began, two years since, to experience symptoms of locomotor ataxy, which commenced and increased in severity almost imperceptibly. The effects of the disease appear to be confined to the muscles of the legs, which seemed to lose their power of acting co-ordinately. The surface possesses its normal sensitiveness ; the muscles their full strength, judging from the manner in which he can use them while lying down. In walking, the motions of the limbs are similar to those of an intoxicated person. He is obliged to fix his eyes upon the place where he wishes to plant his feet, in order to walk with any degree of security. This even requires, apparently, great effort of will. On closing the eyes, or in walking in the dark, he loses confidence and falls easily. The patient was able to attend his ordinary business.

Dr. H. stated that, according to the best authorities, the disease was accompanied with different symptoms in different cases. Sometimes, though not often, the muscles of the arms were affected; there was pain and partial loss of feeling in the limbs; occasionally, the symptoms were confined to one arm and one leg; usually, however, to both legs. In some cases, the special senses, particularly the sight and hearing, were impaired. The limbs do not generally become atrophied, as in ordinary paralysis.

The disease seems to be a degeneration of the posterior columns of the spinal cord; the nerve fibres appear to be so changed that the power of communication between the brain and the muscles of the pelvis and the lower limbs exists in a very abnormal condition. This is owing to atrophy of the nerve fibres which compose the spinal cord.

Dr. Ross gave the history of a case of this disease, which had fallen under his observation. The paper will be given in full in our next issue.

Dr. Bluthardt read a synopsis of the opinions of distinguished authorities in reference to this disease.

Remarks were made upon the general subject by Drs. Paoli, Hildreth, Lyman, Peterson and Durham.

